# 301. Clinical impact of a *Streptococcus pneumoniae* PCR assay on pleural fluid specimens for children with complicated pneumonia

**DOI:** 10.1093/ofid/ofae631.091

**Published:** 2025-01-29

**Authors:** Erin C Ho, Kaitlin E Olson, Molly Butler, Meghan C Birkholz, Kristen Miller, Sarah A Jung, Edwin J Asturias, Samuel R Dominguez

**Affiliations:** University of Colorado School of Medicine, Aurora, CO; University of Colorado School of Medicine, Aurora, CO; Children's Hospital Colorado, Aurora, Colorado; Children's Hospital Colorado, Aurora, Colorado; University of Colorado School of Medicine, Aurora, CO; Children's Hospital Colorado, Aurora, Colorado; University of Colorado School of Medicine, Aurora, CO; University of Colorado School of Medicine, Aurora, CO

## Abstract

**Background:**

While *Streptococcus pneumoniae* (Spn) is the predominant pathogen in pediatric complicated community acquired pneumonia (cCAP), it is infrequently recovered by culture-based methods. Without Spn or another pathogen detected, patients often remain on inappropriately broad-spectrum therapy. We studied the real-world clinical impact of a newly implemented, lab developed Spn PCR assay, with target genes *lytA*, *piaB*, SP2020, for pleural fluid specimens.

**Figure:**
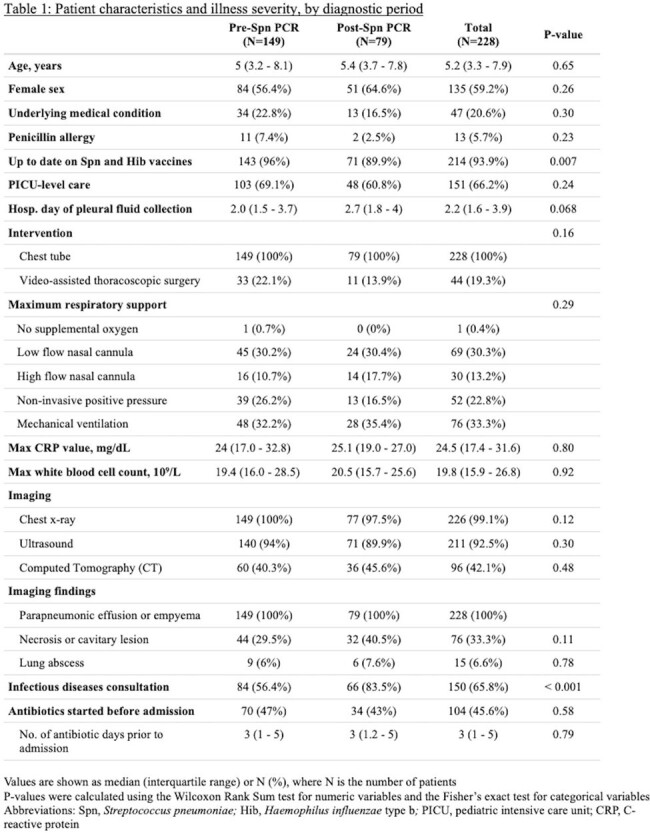

**Methods:**

We compared pathogen detection, antibiotic usage, and outcomes in children hospitalized with cCAP requiring pleural effusion or empyema drainage at Children’s Hospital Colorado between 2016 – 2023. The study population was divided into two diagnostic periods: pre-Spn PCR (October 1, 2016 – July 26, 2022) and post-Spn PCR (July 27, 2022 – September 30, 2023). Kaplan Meier curves and log-rank tests compared time from hospital admission to pathogen detection, optimal therapy, and MRSA therapy discontinuation between periods. Optimal therapy was pre-defined as pathogen-directed therapy or per institutional guidelines (ampicillin in most cases with no pathogen).

**Figure:**
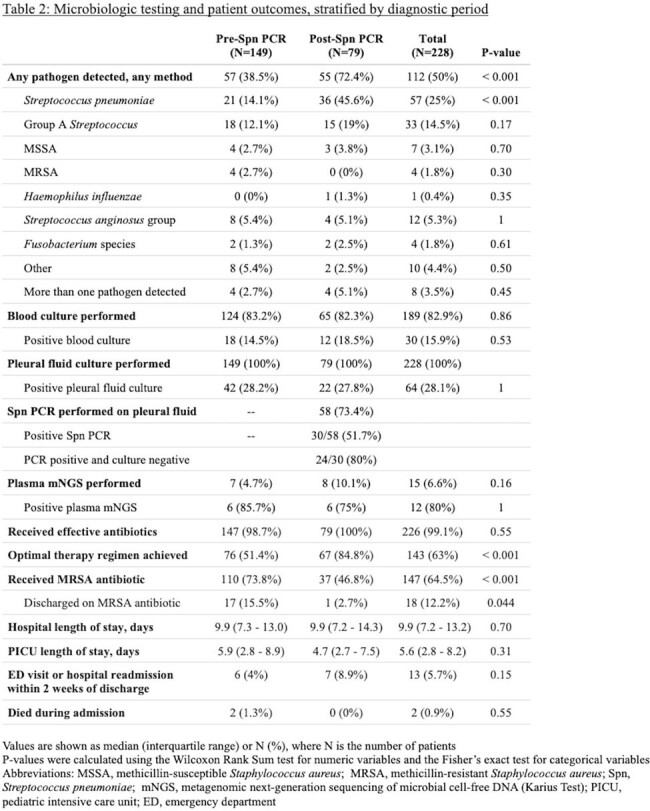

**Results:**

Patient characteristics and illness severities were similar across the pre-Spn PCR (N=149) and post-Spn PCR (N=79) periods, and pleural fluid drainage occurred most frequently on hospital day 2 (Table 1). The post-Spn PCR cohort had a higher percentage with any pathogen detected (72.4% vs. 38.5%, p < 0.001), driven by more Spn detections (45.6% vs. 14.1%, p < 0.001) (Table 2). Time to pathogen detection was shorter in the post-Spn PCR period (p < 0.001, Figure 1). Post-Spn PCR patients were more likely to reach optimal antibiotic therapy (84.8% vs. 51.4%, p < 0.001), with shorter median time to optimal therapy (5.0 days vs. 10.3 days, p < 0.001, Figure 2A) and shorter median time to MRSA antibiotic discontinuation (1.5 days vs. 2.5 days, p = 0.03, Figure 2B). There were no differences in hospital length of stay or readmissions (Table 2).

**Figure:**
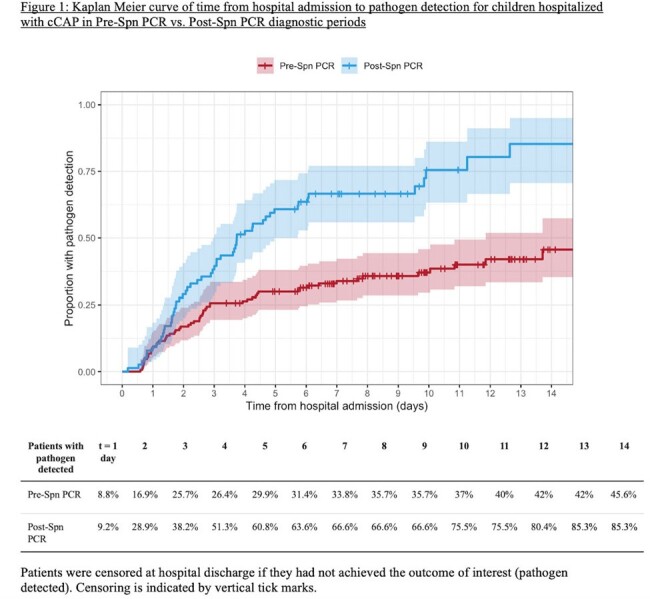

**Conclusion:**

Molecular detection of Spn in pleural fluid of children with cCAP resulted in a significant increase in causative pathogen identification, optimization of antibiotics, and decreased exposure to MRSA antibiotics, demonstrating that PCR is a clinically impactful addition to conventional pneumonia diagnostics.

**Figure:**
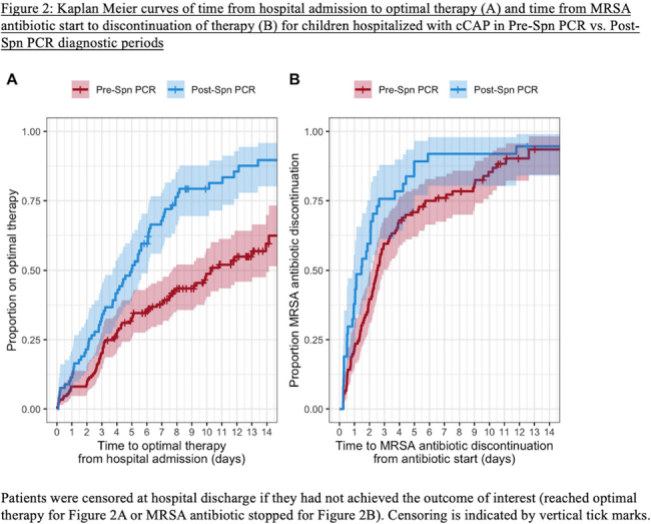

**Disclosures:**

**Molly Butler, PhD**, Pfizer: Conference Attendance **Sarah A. Jung, PhD**, Cobio Diagnostics: Advisor/Consultant|Karius Inc: Advisor/Consultant|Roche Diagnostics: Advisor/Consultant **Edwin J. Asturias, MD**, BioFire: Grant/Research Support|Hillevax: Advisor/Consultant|Merck: Advisor/Consultant|Moderna: Advisor/Consultant|Pfizer: Grant/Research Support **Samuel R. Dominguez, MD, PhD**, BIofire Diagnostics: Advisor/Consultant|BIofire Diagnostics: Grant/Research Support|DelveBio: Grant/Research Support|Karius: Advisor/Consultant|Karius: Grant/Research Support|Pfizer: Grant/Research Support

